# Phenotypic and Genotypic Characterization of Veterinary *Vibrio cincinnatiensis* Isolates

**DOI:** 10.3390/microorganisms8050739

**Published:** 2020-05-15

**Authors:** Claudia Jäckel, Jens Andre Hammerl, Huynh-Huong-Thao Arslan, Cornelia Göllner, Nicole vom Ort, Karin Taureck, Eckhard Strauch

**Affiliations:** 1 Department of Biological Safety, German Federal Institute for Risk Assessment, Max-Dohrn-Str. 8-10, D–10589 Berlin, Germany; claudia.jaeckel@bfr.bund.de (C.J.); jens-andre.hammerl@bfr.bund.de (J.A.H.); thao.arslan@googlemail.com (H.-H.-T.A.); cornelia.goellner@bfr.bund.de (C.G.); Nicole.vomOrt@bfr.bund.de (N.v.O.); 2 Landesuntersuchungsanstalt für das Gesundheits- und Veterinärwesen Sachsen, 01099 Standort Dresden, Germany; ktaureck@gmx.de

**Keywords:** *Vibrio cincinnatiensis*, veterinary isolates, whole-genome sequences, average nucleotide identity, MALDI-TOF MS, antimicrobial resistance

## Abstract

*Vibrio cincinnatiensis* is a halophilic species which has been found in marine and estuarine environments worldwide. The species is considered a rare pathogen for which the significance for humans is unclear. In this study, nine veterinary isolates were investigated that were obtained from domestic animals in Germany. The isolates were mostly recovered from abortion material of pigs, cattle, and horse (amnion or fetuses). One isolate was from a goose. A human clinical strain from a case of enteritis in Germany described in the literature was also included in the study. Whole-genome sequencing (WGS) of all isolates and MALDI-TOF MS (matrix-assisted-laser-desorption/ionization time-of-flight mass spectrometry) were performed to verify the species assignment. All strains were investigated for phenotypic traits including antimicrobial resistance (AMR), biochemical properties, and two virulence-associated phenotypes (hemolytic activity and resistance to human serum). WGS data and MS spectra confirmed that all veterinary isolates are closely related to the type strain *V. cincinnatiensis* NCTC12012. An exception was the human isolate from Germany which is related to the other isolates but could belong to another species. The isolates were similar in most biochemical phenotypes. Only one strain showed a very weak hemolytic activity against sheep erythrocytes, and serum resistance was intermediate in two strains. AMR phenotypes were more variable between the isolates. Resistances were observed against ß-lactams ampicillin and cefoxitin and against tetracycline and the sulfonamide antibiotics trimethoprim and sulfamethoxazole. Some acquired AMR genes were identified by bioinformatics analyses. WGS and MALDI-TOF MS data reveal a close relationship of the veterinary isolates and the type strain *V. cincinnatiensis* NCTC12012, which is a clinical human isolate. As the veterinary isolates of this study were mostly recovered from abortion material (amnions and fetuses), a zoonotic potential of the veterinary isolates seems possible.

## 1. Introduction

*Vibrio cincinnatiensis* is a rare human pathogen. The type strain was isolated from blood and cerebrospinal fluid collected from a 70-year-old male at the University of Cincinnati Hospital in 1986 [1]. The patient suffered from bacteremia and meningitis, and the source of the infection remained undetected. The patient had no known contact with seafood or seawater. In Germany, another case of a suspected *V. cincinnatiensis* infection of an elder immunocompromised patient was reported in 1993 [2]. The strain was isolated from feces of a 67-year-old female suffering from enteritis. Also, in this case, a contact to seawater or an exposure to seafood could not be established.

The species *V. cincinnatiensis* is mostly counted among the human pathogenic representatives of the *Vibrionaceae* (e.g., Reference [3]). However, based on the low number of reports, its status as human enteric or wound pathogen is less clear [4] and its significance as human pathogen remains to be determined [5].

In a few studies on the composition of environmental *Vibrio* communities, the occurrence of *V. cincinnatiensis* in aquatic ecosystems was reported. *V. cincinnatiensis* was found in the Chesapeake Bay, USA, with higher counts in summer months [6], though other *Vibrio* species were more abundant in the same samples. Strains belonging to the *V. cincinnatiensis* were also discovered in inner flesh and liquor obtained from mussels (*Mytilus galloprovincialis*) harvested from the Adriatic Sea of Italy [7]. Another report on the detection of *V. cincinnatiensis* was from the Ohta river of Japan, where strains of the species were recovered in some sampling stations from river water [8]. *V. cincinnatiensis* strains were also isolated of seawater samples from different monitoring points of Lima Sea, Peru [9]. In all studies, *V. cincinnatiensis* strains represented only a small fraction of the resident *Vibrio* populations. However, given the great geographical distances of the investigated regions, it seems likely that the species is present in many aquatic ecosystems throughout the world.

In Germany, *Vibrio* communities were investigated in coastal waters and estuaries in recent years. However, so far, no isolation of *V. cincinnatiensis* strains has been reported [10,11]. In contrast to these results, *V. cincinnatiensis* strains were detected in veterinary specimen obtained from domestic animals in the federal state of Saxony, Germany. The first report of two veterinary *V. cincinnatiensis* isolates were published together with the description of the human isolate in 1993 [2]. The two veterinary isolates originated from stomachs of aborted bovine fetuses and were recovered by an official veterinary laboratory in Dresden, Saxony. The laboratory continued to investigate domestic animals after abortions and could isolate a number of different *Vibrio* spp. strains in parts of the placenta or in aborted fetuses. Parts of these investigations were published in 2002 [12].

This project was undertaken to characterize the veterinary strains, as the role of the species *V. cincinnatiensis* is unclear and its relevance as human pathogen is uncertain. Our laboratory obtained more than 100 suspected *V. cincinnatiensis* isolates from the veterinary laboratory at Dresden that were collected from domestic animals over a period of 20 years. We selected nine representative isolates from different years (1990 to 2011) and different animals to minimize duplicate strains. Most of the strains were from miscarriages of pig and cattle, and one strain was obtained from a goose. The human *V. cincinnatiensis* isolate from the German case [2] was available from the strain collection of the Robert Koch Institute (RKI), Berlin, and was included in the study.

Due to the small number of studies on this species, only a few molecular data are published for *V. cincinnatiensis*. The reference strain NCTC12012 [1] is available from public strain collections, and its whole-genome sequence (WGS) is published (accession UHIE01000001–UHIE01000003). We used this strain to confirm the species identification of the German isolates. Whole-genome sequences of all strains were obtained, and MALDI-TOF MS (matrix-assisted-laser-desorption/ionization time of-flight mass spectrometry) was applied for comparison with the reference.

Furthermore, all strains were characterized for biochemical traits and other phenotypes like hemolytic activity, resistance to human serum, and resistance to antimicrobial agents.

## 2. Materials and Methods

### 2.1. Bacterial Strains

In total, ten *V. cincinnatiensis* isolates from German sources and the reference strain NCTC12012 (other collection strain numbers: ATCC35912, LMG7891, and DSM19608 [1]) were used in this study (Table 1). Nine isolates from domestic animals were obtained from an official veterinary laboratory in Saxony (Landesuntersuchungsanstalt für das Gesundheits- und Veterinärwesen Sachsen, Dresden), and one human strain was from the RKI strain collection [2]. Species identification of all German strains was based on biochemical and other phenotypic characterization.

### 2.2. Mass Spectrometry

Matrix-assisted laser-desorption/ionization time-of-flight mass spectrometry (MALDI-TOF MS) analysis was performed using a Microflex LT system mass spectrometer (Bruker Daltonik, Bremen, Germany) following the manufacturer’s settings. MALDI-TOF MS spectra were obtained by the extraction procedure according to the manufacturer’s protocol as previously described [13]. From every strain, eight spots were applied onto the MALDI target (Bruker Daltonik) and measured three times, resulting in 24 single spectra for each strain. According to the Bruker specifications, low-quality spectra (e.g., outliers) were excluded using flexAnalyses software (version 3.4, Bruker Daltonik). After this quality check, the single spectra of each strain were summarized (at least 20 spectra per strain) and processed using the BiotyperTM software (version 3.1.66) to generate main spectra, each containing the 70 most prominent peaks of the single spectra. For each of the eleven strains (including reference strain NCTC12012), main spectra profiles (MSP) were created. Spectra preprocessing parameters included mass adjustment, smoothing, baseline correction, normalization, and peak picking using the default settings of the BiotyperTM software. MALDI-TOF MS results interpretation was done according to the Bruker score system.

### 2.3. Whole-Genome Sequencing

WGS was performed with all German strains. Preparation of genomic DNA and short-read whole-genome sequencing (Illumina MiSeq, San Diego, CA, USA) was conducted as previously described [14]. SPAdes de novo assemblies of raw reads were performed using the PATRIC (Pathosystems Resource Integration Center) database (release 3.5.37) [15] following submission to the automated Prokaryotic Genome Annotation Pipeline of the National Center for Biotechnology Information website for genome annotation. Identification and assessment of putative prophage sequences was performed using the PHAge Search Tool (PHAST) according to the recommendations of the providers [16]. To detect specific genetic features within the genome sequences, different in silico analysis tools of the Center for Genomic Epidemiology (CGE), provided by the Danish Technical University, were used. Initial plasmid prediction was performed with the PlasmidFinder Web tool (release 2.0) [17]. CGE-based analyses were performed by using de novo assemblies of genomes. For in silico predictions, a minimum identity level of 50% (PlasmidFinder) as well as a coverage level of at least 20% was used.

### 2.4. Data Availability

Genome sequences of *V. cincinnatiensis* isolates have been deposited in GenBank at the National Center for Biotechnology Information (NCBI) under the accession numbers given in Dataset S1 (bioproject no’s PRJNA521612–PRJNA521622; biosample no’s SAMN10907307–SAMN10907317, and accession no’s SGOM00000000–SGOW00000000) (Supplementary Table S1).

### 2.5. Bioinformatic Analyses

Average nucleotide identity (ANI) is a tool to determine if two genomes belong to the same species. All genome sequences of the German strains were compared pairwise to the published genome of the type strain NCTC12012 (accession UHIE01000001–UHIE01000003). The ANI online calculation tool http://enve-omics.ce.gatech.edu/ani/ was used with the default settings (alignment options: minimum length 700 bp, minimum identity 70%, minimum alignments 50; fragment options: window size 1000 bp, and step size 200 bp).

To determine the phylogenetic relationship of the isolates, a CSIPhylogeny (version 1.4; https://cge.cbs.dtu.dk/services/CSIPhylogeny/) tree based on single nucleotide polymorphism (SNP) was prepared. The web-based tool was used under default settings and the exclusion of heterozygous SNPs. As reference the genome of strain NCTC12012 (accession UHIE01000001–UHIE01000003) was used. Nucleotide variations were predicted according to the specifications provided by Kaas et al. [18].

Multilocus sequence analysis (MLSA) was based on the PUBMLST scheme for *Vibrio* spp. (https://pubmlst.org/vibrio/) [19]. Sequences of housekeeping genes (*atpA, gyrB, pyrH,* and *recA*) were retrieved from WGS data by using BlastN (https://blast.ncbi.nlm.nih.gov/Blast.cgi). Concatemers were created using Accelrys Gene version 2.5 (Accelrys Inc., San Diego, CA, USA). The evolutionary history was inferred using the neighbor-joining method, and bootstrap test (1000 replicates) was performed. The evolutionary distances were computed using the Kimura 2-parameter method. All positions containing gaps and missing data were eliminated. Evolutionary analyses were conducted in MEGA7 [20].

For the detection of antimicrobial resistance (AMR) genes, the ResFinder web tool (release 3.1.0, https://cge.cbs.dtu.dk/services/ResFinder/) was used, which identifies acquired AMR genes and/or chromosomal mutations in total or partial sequenced isolates of bacteria. Analyses were performed by using de novo assemblies of genomes. For in silico predictions of acquired AMR genes, a minimum identity level of 30% of the coding sequences as well as a coverage level of at least 20% of the coding length was used for the initial search.

For the detection of *Vibrio*-specific virulence determinants, the MyDbFinder Web tool (release 1.1) was used with a database of the virulence factor database (VFDB; http://www.mgc.ac.cn/VFs/) [21]. CGE-based analyses were performed by using de novo assemblies of genomes. For in silico predictions, a minimum identity level of 30% (MyDbFinder) as well as a coverage level of at least 20% was used. To screen for *V. cincinnatiensis*-specific gene variants of the latter and of additional virulence determinants, the segmented genome fragments were applied to the BLASTN search of the NCBI database and were compared to selected reference sequences using default settings.

### 2.6. Phenotypic Characterization

Strains were phenotypically characterized by biochemical tests used in routine diagnostics. Each strain was inoculated into 0.85% NaCl, and turbidity was adjusted to 0.5 McFarland standard (bioMerieux, Marcy-l’Etoile, France). Tests included growth in 1% peptone water with 0%, 2%, 6%, and 10% NaCl and cytochrome oxidase. Lysine decarboxylase, arginine dihydrolase, ornithine decarboxylase, nitrate reductase, and utilization of a number of carbohydrates were performed with the API 20NE and API 20E systems (bioMerieux). The test strips were incubated either at 29 °C (API 20NE) or 37 °C (API 20E) and read at 24 and 48 h. Quality control testing was performed with every test.

### 2.7. Hemolytic Activity

Blood agar plates were prepared with Mueller Hinton agar (Oxoid GmbH, Wesel, Germany) supplemented with 1% NaCl and contained 4% sheep erythrocytes (BfR, Berlin, Germany) or 4% human erythrocytes (German Red Cross, blood donation service, Berlin Wannsee, Germany). Erythrocytes were washed three times in cold phosphate buffered saline and pelleted for 5 min at 400× *g* and 10 °C before use.

Prior to hemolysis assay, bacteria were cultivated from glycerol stocks on Mueller Hinton agar plates overnight at 37 °C; 4 mL Mueller Hinton broth was inoculated with one single colony and incubated for 12 to 14 h at 37 °C with constant shaking (200 rpm). All culture media were supplemented with 1% NaCl. Hemolysis was also tested on Columbia agar with sheep blood (Oxoid, Basingstoke, UK).

In order to investigate the hemolytic activity of the strains, 10 µL of the overnight cultures was spotted on a blood agar plate and incubated at 37 °C to obtain macrocolonies. Zones of hemolysis around the macrocolonies were visually controlled and scored for up to 72 h [14]. All experiments were performed twice.

### 2.8. Serum Resistance

The resistance test against human serum was carried out as described previously [22,23]. Logarithmic growing bacteria were transferred to 96-well plates containing 100 µL peptone-glucose-broth (1% glucose, 0.0075% bromothymolblue, 1% peptone, 0.5% NaCl, pH 7.4) with different concentrations of human serum (0%, 10%, 20%, 40%, 60%, and 80% pooled serum obtained from healthy volunteers). Plates were incubated at 37 °C for 24 h, and serum resistance was determined by examining the color changes from blue to yellow indicating bacterial growth. Experiments were performed twice with each strain. Strains of *E. coli* K-12 and *E. coli* K-12 (pKT 107) [24] carrying the pKT107 serum resistance plasmid were used as negative and positive controls, respectively. Isolates able to grow in the presence of 60–80% human serum were classified as serum resistant, while growth in the presence of 20–40% and 0–10% was designated as intermediate and sensitive, respectively.

### 2.9. Antimicrobial Resistance Testing

Susceptibility testing to antimicrobial agents was conducted by broth microdilution according to the guidelines of the Clinical and Laboratory Standards Institute (CLSI) [25] using custom-defined microtiter plates (EUVSEC and EUVSEC2, Trek Diagnostic Systems, East Grinstead, United Kingdom). Tests were performed using the following antimicrobial substances: ampicillin, azithromycin, cefepime, chloramphenicol, ciprofloxacin, colistin, ertapenem, cefotaxime, cefoxitin, gentamicin, imipenem, meropenem, nalidixic acid, sulfamethoxazole, cefotaxime/clavulanic acid, ceftazidime, ceftazidime/clavulanic acid, temocillin, tetracycline, tigecycline, and trimethoprim. The selection of agents corresponds to the harmonized panel of antimicrobials, which is used for monitoring *E. coli* and *Salmonella* in the European Union [26].

Susceptibility tests were performed using Mueller–Hinton (MH) agar and cation-adjusted MH broth (Thermo Fisher Scientific, Braunschweig, Germany) without supplementation of additional sodium chloride. Minimal inhibitory concentrations (MIC) and cutoff values for the interpretation of the results are provided in Supplementary Table S2. *Escherichia coli* ATCC25922 was used as reference standard and quality control. In general, results were interpreted according to CLSI clinical breakpoints specific for *Vibrio* spp. [25].

## 3. Results and Discussion

### 3.1. Species Identification Applying MALDI-TOF MS

Nine veterinary *V. cincinnatiensis* isolates, one German clinical isolate, and the type strain NCTC12012 were used in this study (Table 1). To confirm the species determination, in a first step, MALDI-TOF mass spectrometry was used, as this technique has been shown to be a reliable diagnostic tool for identification of *Vibrio* species [13]. For all investigated strains (Table 1) including the reference strain NCTC12012, main spectra (MSP) were generated. The MSPs of the German strains were pairwise matched against the MSP of the reference strain NCTC12012 using the Bruker software Biotyper 3. The scoring system (Table 2) indicates a highly probable species identification as *V. cincinnatiensis* for all strains (score > 2.3). The only exception is the human strain 19-VB00020 with a lower score value of 2.149. This score is interpreted as secure genus identification and probable species identification.

### 3.2. Species Identification Using Average Nucleotide Identity (ANI)

A taxonomic affiliation of bacterial genomes can be performed by using ANI, which is a similarity index between genomes [27]. An ANI value greater than 95% between two genomes indicates that the two genomes belong to the same bacterial species (http://enve-omics.ce.gatech.edu/ani/).

The genomes of all German strains were obtained using short-read whole-genome sequencing (Illumina). WGS revealed that the genome sizes of the sequenced strains vary between 3.5–4.0 Mbp and that the number of putative coding sequences (CDS) was between 3.3–4.0 × 10^3^. More detailed information of the genomes is given in Supplementary Table S1. An ANI calculation was performed between each genome of the German strains and the genome of the reference strain NCTC12012. The calculated ANI values for all veterinary strains were around 99%, confirming their taxonomic affiliation to the species *V. cincinnatiensis* (Table 2). The only exceptional ANI value was obtained in case of the calculation based on the comparison of the genomes of strains 19-VB00020 and NCTC12012. In this case, the ANI value was only 88.7%. Interpretation of the MALDI-TOF MS results and ANI calculation suggest that the German human pathogenic strain [2] is related but does not belong to the species *V. cincinnatiensis.*

### 3.3. Phylogenetic Relationship Based on Single Nucleotide Polymorphism (SNP)

To characterize the relationship between the investigated strains further, an SNP analysis was performed. All sequences were mapped to the NCTC12012 genome as reference and screened for relevant nucleotide variations [18]. In the analysis included were four *V. cincinnatiensis* genomes deposited by the Centers for Disease Control and Prevention (CDC), Atlanta, USA.

In total, the percentage of the reference genome covered by all isolates was 66.6% (2,530,612) positions in all analyzed genomes. The number of SNPs between the strains varies between 3 to 30,935 (Supplementary Table S3) with the genome of strain 19-VB00020 (human strain) distinctly differing from all other genomes (range of SNPs of this genome between 24,040 to 30,935 to the remaining strains).

**Figure 1:**
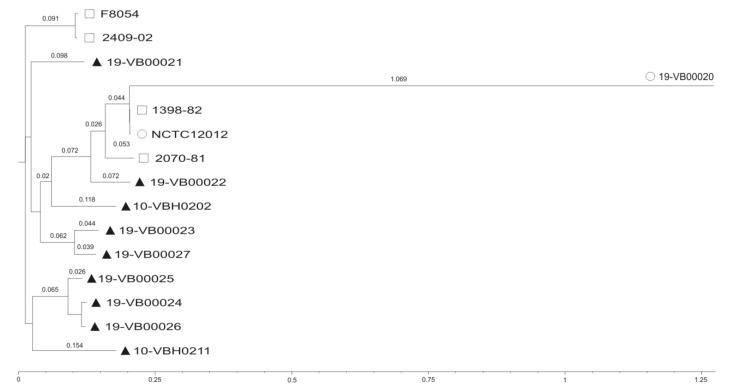
Single nucleotide polymorphism (SNP)-based phylogeny tree of *Vibrio cincinnatiensis* strains: veterinary isolates (▲), human isolates (О), or isolates of unknown origin (□). SNP tree was conducted using CSIPhylogeny 1.4 under default settings and the exclusion of heterozygous SNPs. Single nucleotide polymorphisms (SNPs) were called by mapping to the *V. cincinnatiensis* NCTC12012 genome as reference. Scale bar represents the number of nucleotide substitutions per site and numbers indicate branch length (accession numbers for strain F8054: CP046848 and CP046849; for 1398-82: CP046815 and CP046816; for 2070-81: CP046802 and CP046801; and for 2409-02: CP035697 and CP035698).

Using the concatenated alignments of high-quality SNPs, maximum likelihood trees were created using FastTree [28] (Figure 1).

The length of the branches displays the distinct separation of the human isolate 19-VB00020 to all other strains including the reference strain NCTC12012. The veterinary strains differ from each other (SNP differences between 778 to approx. 12,000), indicating that they are not closely related. This reflects probably the selection of bacteria. Isolates from different years and different animals were chosen to achieve a higher diversity and to minimize potential replicates. To obtain more information on possible phylogenetic relationships of the German human strain 19-VB00020, the PATRIC bioinformatics tool “similar genome finder” (https://docs.patricbrc.org/user_guides/services/similar_genome_finder_service.html) was applied. Using default settings, no related genome was detected; by reducing the stringency of the search, the most similar genome was that of reference strain *V. cincinnatiensis* NCTC12012 (data not shown).

### 3.4. Multilocus Sequence Analysis (MLSA) of Housekeeping Genes

To study the phylogenetic relationship of the human strain 19-VB00020 to the remaining strains, we constructed a core genome by applying the *Vibrio*-MLSA scheme available on the PubMLST website [19]. The sequences of four housekeeping genes (*atpA, gyrB*, *pyrH*, and *recA)* were extracted from WGS data. The sequences were arranged in concatemers of the order *atpA-gyrB*-*pyrH*-*recA* with a length of 1964 bp. We included concatenated sequences of the reference strain NCTC12012 and of type strains from other species (*V. parahaemolyticus* ATCC17802, *V. cholerae* N19961, and *V. vulnificus* ATCC2765) into the phylogenetic analysis. Also, a concatenated sequence of the *V. cincinnatiensis* strain 2409-02, deposited by the Centers of Disease Control, Atlanta, USA, was added to the analysis.

The *recA* gene of two strains (19-VB00022 and 19-VB00023) contained insertions within the 3’ coding region of the *recA* gene, so only 404 bp instead of 462 bp of the *recA* gene were used for the construction of all concatemers (truncation of 3’ sequences of *recA*). Insertions into coding regions of *recA* genes were also observed in a number of *V. parahaemolyticus* strains [29] and make it impossible to assign an allele number and sequence type for those strains.

**Figure 2:**
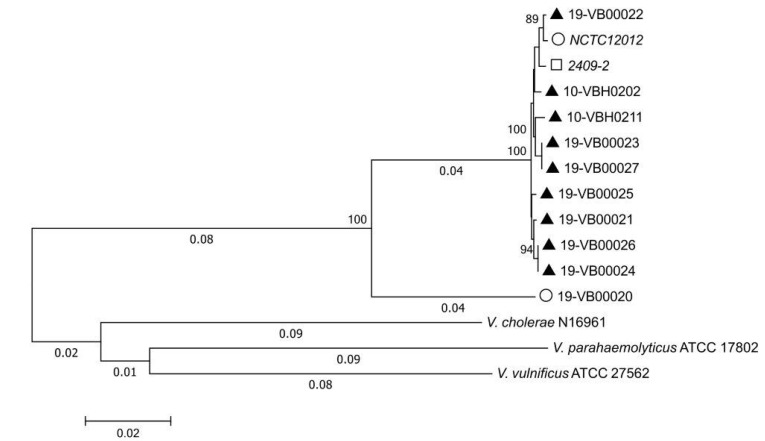
Phylogenetic tree of *V. cincinnatiensis* strains and different *Vibrionaceae* strains based on concatenated sequences in the order *atpA-gyrB-pyrH-recA* (1964 bp) constructed using MEGA7 [20]. Symbols: veterinary strains (▲), human strains (О), and unknown origin (□). The evolutionary history was inferred using the neighbor-joining method. The optimal tree with the sum of branch length = 0.46004156 is shown. The percentages of replicate trees in which the associated taxa clustered together in the bootstrap test (1000 replicates) are shown next to the branches (only values above 70% are shown). The tree is drawn to scale, with branch lengths (next to the branches) in the same units as those of the evolutionary distances used to infer the phylogenetic tree. The evolutionary distances were computed using the Kimura 2-parameter method and are in the units of the number of base substitutions per site. *V. cincinnatiensis* strain 2409-02 was deposited by CDC with accession NZ_CP035697 and NZ_CP035698. *V. cholerae* N16961 accession NC_002505/NC_002506, *V. parahaemolyticus* ATCC17802 accession NZ_MQUE00000000, and *V. vulnificus* accession ATCC27562 accession NZ_CP012881/NZ_CP012882.

The phylogenetic analysis of the concatemers *atpA-gyrB*-*pyrH*-*recA* derived from all strains displayed one cluster containing all veterinary *V. cincinnatiensis* isolates together with the reference strain NCTC12012 and strain 2409-02 for which accession was deposited by CDC (Figure 2). The phylogenetic tree shows that the type strains of *V. cholerae, V. parahaemolyticus*, and *V. vulnificus* are distant and that strain 19-VB000020 is closer related to the *V. cincinnatiensis* strains but does not fall into this cluster. The identity of all concatenated sequences of the *V. cincinnatiensis* cluster is higher than 99%, whereas the sequence similarity of 19-VB00020 to this cluster is approx. 92%.

### 3.5. Antimicrobial Resistance

Antimicrobial resistance testing was conducted by broth microdilution according to the guidelines of the Clinical and Laboratory Standards Institute (CLSI) [25]. The tests were performed using 19 substances and two combinations (see Table S2), where by the selection of agents corresponds to the harmonized panel of antimicrobials, which is used for monitoring *E. coli* and *Salmonella* in the European Union.

Antimicrobial resistances were observed against the ß-lactam antibiotics ampicillin and cefoxitin and against tetracycline and the sulfonamide antibiotics trimethoprim and sulfamethoxazole. In Table 3, the results for selected antimicrobial agents are shown; the complete results of all strains and the interpretation criteria are displayed in Supplementary Table S2. One strain (19-VB00024) exhibited resistance to five antimicrobials, one strain (10-VBH00211) was resistant against two antimicrobials, and two strains (19-VB00023 and 19-VB00026) were resistant against one antimicrobial agent.

For some of the tested antimicrobials, e.g., nalidixic acid, CLSI interpretation criteria are not available for *Vibrio* spp.; in the case of nalidixic acid, it is an interesting observation that one strain (10-VBH0202) shows a remarkably increased resistance compared to all other strains. The resistance phenotype of this strain may be caused by a mutation of the *gyrA* gene leading to an amino acid substitution (serine to isoleucine) in position 83 that was described in other *Vibrio* species [30].

The bioinformatic search detected acquired antimicrobial resistance genes and/or chromosomal mutations only in the genomes of three strains (Table 3). The gene *qnrVC6* coding for a quinolone resistance protein [31] was found in strain 19-VB00021. Bacteria carrying *qnr* genes show reduced susceptibility to ciprofloxacin, and in combination with mutations in other genes, full quinolone resistance can emerge. This can contribute to a decreased efficiency of quinolones in antibiotic treatment. So far, many *qnrVC* alleles have been found in the *Vibrionaceae* family and among environmental aquatic-borne species [31].

Strain 19-VB00024 showed resistance to five antimicrobials. While no acquired ß-lactamase genes were detected, two resistance genes for sulfonamide antibiotics (*sul2* encoding a sulfonamide-resistant dihydropteroate synthase and *dfrA1* encoding a dihydrofolate reductase) were identified. The *dfrA1* gene lies upstream of an *aadA1* gene coding for an aminoglycoside nucleotidyltransferase. The two genes seem to be part of an integron, as an integron integrase gene (*intI2*) is adjacent to *dfr1* (332 bp upstream of *dfrA1*). Three other resistance genes of this strain are located on a small contig. Two other aminoglycoside phosphotransferase genes *aph(3″)-Ib* and *aph(6)-Id* are head to tail arranged, and a *tetB* gene coding for a tetracycline efflux pump is located on the same contig. Phenotypically, no resistance of strain 19-VB00024 against gentamycin, the only aminoglycoside antibiotic tested, was observed despite the presence of three aminoglycoside resistance genes. The strain possessed an intermediate resistance level against tetracycline and an intermediate resistance against chloramphenicol. The latter resistance may be caused by a chloramphenicol exporter protein encoded by the *floR* gene.

Strain 10-VBH0211 possessed fewer AMR genes than strain 19-VB00024 (Table 3). Despite the presence of two aminoglycoside resistance genes *aph(3″)-Ib* and *aph(6)-Id* (which are arranged head to tail), the strain was susceptible to gentamycin. All other AMR genes of the strain are located on separate contigs.

Most studies on AMR genes of *Vibrionaceae* have been conducted in *V. cholerae* [32,33]. It has been shown that AMR genes are located in cassettes on mobile genetic elements like ICEs (integrative conjugative elements), integrons, transposons, or plasmids. Clustering of AMR genes is observed in strain 19-VB00024, indicating that, in *V. cincinnatiensis*, AMR genes may be part of such mobile elements. The majority of integron cassettes are known to be promoterless, and AMR genes may be poorly expressed [34]. This could explain why gentamycin resistance was not observed in strains 10-VBH0211 and 19-VB00024 though the arrangement of most AMR genes in the two strains is unknown.

### 3.6. Phenotypic Characteristics

The human and the veterinary strains had originally been identified as *V. cincinnatiensis* by phenotypical characterization. We performed a number of tests to compare the human strain 19-VB00020 to the remaining *V. cincinnatiensis* strains (Supplementary Table S4) The biochemical reactions were mostly identical between the strains. All strains did not grow without NaCl and were able to grow at a concentration of 6% with one exception (strain 10-VBH0211). All strains were oxidase positive, reduced nitrate to nitrite, and used *myo* inositol, as had been described earlier [3,35]. The human strain from Germany was positive for lysine decarboxylation, whereas in all other strains the reaction was negative. This phenotype was described as variable before [2,35]. All strains grew on thiosulfate-citrate-bile salts-sucrose (TCBS) agar with a yellow color and on *Vibrio* chrome agar, which is nowadays an important medium for isolation of *Vibrio* spp., colonies of all investigated strains were colorless. It was reported that, in comparison to other *Vibrio* species, *V. cincinnatiensis* isolates grow poorly on TCBS medium, which is commonly used for primary selection [3,35]. However, most strains of this study grew well within 24 h on TCBS at 37 °C and on complete media (e.g., liquid broth).

### 3.7. Phenotypes Associated with Virulence

Potentially, human pathogenic vibrios often grow on blood agar displaying hemolytic activity to erythrocytes [3]. We investigated all German strains on agar plates containing sheep erythrocytes or human erythrocytes. In our hands, most strains did not show any hemolytic activity up to an incubation time of 72 h (Table 4). Only one strain (19-VB00022) showed a weak hemolytic zone on plates containing human erythrocytes after 72 h of incubation. Positive controls in our hemolysis tests were strains of *V. cholerae* non-O1, non-O139, and *V. metschnikovii*, which developed clearly visible hemolysis zones within 24 h of incubation. Strain 19-VB00020, the human isolate from Germany, had been tested negative for hemolysis before [2]. Cell free supernatants of Japanese isolates of *V. cincinnatiensis* (three isolates) were reported to show hemolytic activity against rabbit erythrocytes [8].

Another virulence-associated phenotype of bacteria is resistance to human serum which enables bacteria to escape the bacteriocidal effects of serum and to survive in blood. It is well known that clinical strains of *V. vulnificus* are more resistant to human serum than environmental strains of this species [36]. We investigated the German strains for this phenotype; however, only two out of ten strains showed an intermediate resistance level (Table 4) and the remaining strains were sensitive. Similar investigation on *V. cholerae* non-O1, non-O139 [37], *V. vulnificus* [22], and *V. parahaemolyticus* [38] had revealed that a significant number of strains exhibited serum resistance under these conditions.

The results of the investigated phenotypic tests do not support a potential pathogenicity of the strains for humans or animals. However, given the source of most veterinary strains as well as the human strain from inner organs or fetuses, it must be assumed that survival within the hosts is possible by defying host defense mechanisms.

### 3.8. Bioinformatic Search for Putative Virulence Factors

*V. cincinnatiensis* is a natural inhabitant of aquatic ecosystems and has to fight biotic and abiotic stress in aquatic environments, to defend against competitors, and to adapt to specific niches. This means that many traits evolved in response to challenges in the natural environment. Such traits may be regarded as virulence-associated factors when strains enter a human host [39]; however, only experimental approaches would corroborate a function as virulence factor in a mammalian host.

A bioinformatic analysis was performed using the virulence factor database (VFDB) to search for putative virulence factors of the *V. cincinnatiensis* strains [21]. This data set yielded results showing the presence of some genes encoding chemotaxis proteins (*cheY* and *cheR*) and flagella genes in all strains. Chemotaxis and motility can contribute to virulence in animal models, as was shown in *V. cholerae* [40,41]. Both traits are also necessary for survival in the natural environment [39]. A gene encoding a protein related to the cholix toxin of *V. cholerae* strains which is eukaryotic elongation factor 2-specific ADP-ribosyltransferase toxin was not detected in our *V. cincinnatiensis* strains [42].

BLAST searches of the annotated genomes revealed that genes coding for quorum sensing autoinducer synthases are found in all *V. cincinnatiensis* genomes derived. In *V. cholerae*, quorum sensing is involved in regulation of virulence gene expression [39,43], but quorum sensing is also a widespread mechanism of many environmental bacteria to monitor their cell population density. All *V. cincinatiensis* genomes harbor genes encoding the autoinducer synthases CsqA and LuxS, which are producers of the autoinducers-1 (CAI-1) and autoinducers-2 (AI-2) in *V. cholerae* [43]. The coding sequence of a *hapR* gene encoding a quorum sensing-controlled negative regulator was also identified in all genomes [44]. Secretion systems play a major role in virulence of *Vibrio* species. Type III secretion systems (T3SS) are found in *V. cholerae* non-O1, non-O139, and *V. parahaemolyticus* [45,46,47] and contribute to virulence in a human host. While the bioinformatics analysis did not reveal any genes of T3SS in the *V. cincinnatiensis* genomes, genes coding for type VI secretion systems (T6SS) were detected in all strains. T6SSs play a role in the inflammation process in eukaryotic hosts but are also important in the natural aquatic environment for survival [39,48]. T6SSs are involved in protection against predators and competition against antagonistic microorganisms.

Though phenotypically no hemolysis was detected, all strains harbor a *hlyIII* gene encoding hemolysin III. The hemolysin III of *V. vulnificus* showed hemolytic activity in crude extracts of *E. coli* after cloning of the *hlyIII* gene [49]. In an animal model, a *hlyIII* mutant of *V. vulnificus* was attenuated compared to the wild type, which indicates that the hemolysin contributes to virulence [49,50]. However, when plating wild type and isogenic mutant on blood agar plates, no difference in hemolytic activity was observed. It was discussed that the gene may be not expressed under the experimental conditions [49], which may be also the case in the investigated *V. cincinnatiensis* strains. The annotation of the genomes indicates the presence of more hemolysin genes; however, their relevance as virulence factors must be proven experimentally.

## 4. Conclusions

*V. cincinnatiensis* was rarely described in the literature, though the few reports in which this species was studied suggest that strains of the species occur worldwide in coastal and marine waters. The studies indicate that *V. cincinnatiensis* comprises only a small part of the *Vibrio* communities [6,7,8].

The species is regarded as a potential human pathogen, but the number of reported cases in the literature is low [1,2]. According to an older review [35], six human isolates and six non-marine animal isolates had been studied in the *Vibrio* Reference Laboratory of the CDC up to 2006. The German veterinary strains are closely related to the human pathogenic type strain NCTC12012 and are true members of the species *V. cincinnatiensis.* In contrast, the clinical strain from a German patient [2] may belong to another related but not yet defined *Vibrio* species.

During the investigations from 1990 to 2011, the Saxonian veterinary lab recovered more than 100 isolates of *V. cincinniatensis* from domestic animals in the federal state of Saxony of Germany. It is surprising that the animals had come into contact with these bacteria, as the species is regarded as a halophilic inhabitant of coastal and marine ecosystems. No thorough investigation into the possible source of the bacteria was undertaken. An uptake through food from marine origin was regarded as unlikely but could not be ruled out. Therefore, a satisfactory explanation for their presence in these animals cannot be given. Similar findings on *Vibrio* sp. in domestic animals were also published by two other official German veterinary laboratories from Thuringia and Bavaria [51,52]. In a recent study, amplicon sequencing of 16S rRNA genes derived from material of placenta and fetal abomasum of cattle revealed in few cases the presence of *Vibrio* DNA sequences [53], but most of the identified bacterial DNA belonged to different taxonomic genera.

It is possible that *V. cincinnatiensis* may be present in inland areas where brackish lakes or rivers occur. Infections with other *Vibrio* species (*V. vulnificus*) in inland areas have been reported [54]. Occurrence of *V. navarrensis* strains in low salinity aquatic environments away from the coast [55] in rivers and sewage was also reported. Studies in Austria have shown that *V. cholerae* non-O1, non-O139 is found in alkaline seas of central Europe and migratory birds can act as vectors in long-distance transport of these bacteria [56]. As *V. cincinnatiensis* can grow in low saline concentrations [8], it seems possible that it may occur in inland waters of Germany and that the animals ingested the bacteria by uptake of surface water.

Our phenotypic studies on virulence associated traits do not provide support for a pathogenicity potential of the strains, and the possibility remains that the bacteria were only commensals. It should be noted, however, that many isolates were obtained from abortion material and some even from inner organs of fetuses. These sources indicate a potential pathogenicity. Additionally, as the veterinary isolates are closely related to the human pathogenic strain NCTC12012, a zoonotic potential seems possible. The “One Health” concept acknowledges that human, animal, and environmental health are interrelated. Further research should aim to identify the reservoirs or sources and the ways of transmission of this species in order to elucidate a possible role as a zoonotic agent.

## Figures and Tables

**Table 1:** *V. cincinnatiensis* strains used in this study.

Strain ID	Host	Source	Year of Isolation
19-VB00020 *	human	gut (diarrhea)	1987
19-VB00021	cattle	fetus abomasum	1990
19-VB00022	goose	heart, brain	2001
19-VB00023	pig	fetus, amnion	2008
19-VB00024	pig	amnion	2010
19-VB00025	horse	fetus, amnion	2010
19-VB00026	cattle	abomasum	2010
19-VB00027	pig	amnion	2011
10-VBH0211	pig	fetus, amnion	1999
10-VBH0202	pig	fetus, amnion	2010
NCTC12012	human	reference strain	1986

* Strain H388/87 in reference [2].

**Table 2:** Species identification: Results of MALDI-TOF MS (matrix-assisted-laser-desorption/ionization time-of-flight mass spectrometry) and average nucleotide identity (ANI) by comparison with the reference strain NCTC12012.

Strain	Log Score (MALDI-TOF) *	ANI (%) **	Species
19-VB00020	2.149	88.83%	*Vibrio* sp.
19-VB00021	2.740	99.07%	*V. cincinnatiensis*
19-VB00022	2.658	99.36%	*V. cincinnatiensis*
19-V1B00023	2.601	99.18%	*V. cincinnatiensis*
19-VB00024	2.599	99.03%	*V. cincinnatiensis*
19-VB00025	2.636	99.10%	*V. cincinnatiensis*
19-VB00026	2.628	99.06%	*V. cincinnatiensis*
19-VB00027	2.633	99.16%	*V. cincinnatiensis*
10-VBH0211	2.628	98.84%	*V. cincinnatiensis*
10-VBH0202	2.653	98.96%	*V. cincinnatiensis*

* Pairwise comparison of main spectra profiles (MSP) of each strain with MSP of strain NCTC 12012; score > 2.3 highly probable species identification, scores 2.0–2.3 secure genus identification and probable species identification. ** Pairwise comparison to genome of strain NCTC12012 (accession UHIE01000001–UHIE01000003 two-way ANI results) http://enve-omics.ce.gatech.edu/ani/.

**Table 3:** Phenotypic and genotypic results of antimicrobial resistance of *V. cincinnatiensis* strains.

Isolate	AMP	CHL	CIP	FOX	GEN	NAL *	SMX	TAZCLA *	TEMOCI *	TET	TMP	AMR Genes **
19-VB00020	16	≤8	≤0.015	16	≤0.5	≤4	≤8	≤0.12	32	≤2	4	-
19-VB00021	16	≤8	0.06	8	2	≤4	≤8	≤0.12	8	≤2	1	*qnrVC6*
19-VB00022	4	≤8	≤0.015	8	2	≤4	≤8	≤0.12	16	≤2	≤0.25	-
19-VB00023	8	≤8	≤0.015	32	1	≤4	≤8	0.25	16	≤2	1	-
19-VB00024	32	16	0.03	32	2	≤4	1024	0.5	32	64	>32	*aph(3″)-Ib, aph(6)-Id, aadA1,*
												*floR, sul2, tetB, dfr1*
19-VB00025	8	≤8	≤0.015	16	2	≤4	≤8	0.25	16	≤2	0.5	-
19-VB00026	32	≤8	≤0.015	16	≤0.5	≤4	≤8	0.5	32	≤2	0.5	-
19-VB00027	≤1	≤8	≤0.015	8	4	≤4	≤8	≤0.12	2	≤2	≤0.25	-
10-VBH0211	2	≤8	≤0.015	4	≤0.5	≤4	1024	≤0.12	2	8	>32	*aph(3″)-Ib, aph(6)-Id, floR,*
												*sul2, tetB*
10-VBH0202	4	≤8	0.5	16	4	>128	≤8	0.25	8	≤2	1	-
NCTC12012	8	≤8	≤0.015	8	1	≤4	≤8	≤0.12	8	≤2	0.5	-

Dark grey boxes indicate resistance, and light grey boxes indicate intermediate resistance; minimal inhibitory concentration (MIC) is concentration in µg/mL. Interpretation criteria is according to Clinical and Laboratory Standards Institute (CLSI) [25]. Abbreviations: AMP, ampicillin; CHL, chloramphenicol; CIP, ciprofloxacin; FOX, cefoxitin; GEN, gentamicin; NAL, nalidixic acid; SMX, sulfamethoxazole; TAXCLA, cefotaxime/clavulanic acid; TAZCLA, ceftazidime/clavulanic acid; TEMOCI, temocillin; TET, tetracycline; and TMP, trimethoprim. Antimicrobial resistance (AMR) genes derived from genome sequences are *qnrVC6* (coding for quinolone resistance protein), *aph(3″)-Ib* (aminoglycoside phosphotransferase), *aph(6)-Id* (aminoglycoside phosphotransferase), *aadA1* (aminoglycoside nucleotidyltransferase), *floR* (chloramphenicol exporter), *sul2* (sulfonamide resistant dihydropteroate synthase), *tetB* (tetracycline efflux protein), and *dfr1* (dihydrofolate reductase). * No interpretation criteria given by CLSI. ** AMR genes detected by ResFinder tool (Center for Genomic Epidemiology, Lyngby, Denmark).

**Table 4:** Virulence-associated phenotypes, hemolytic activity against sheep and human erythrocytes, and resistance against human serum.

Strain	Hemolysis of Sheep Erythrocytes	Hemolysis of Human Erythrocytes	Resistance to Human Serum **
19-VB00020	-	-	sensitive
19-VB00021	-	-	sensitive
19-VB00022	-	(+) *	sensitive
19-VB00023	-	-	sensitive
19-VB00024	-	-	sensitive
19-VB00025	-	-	intermediate
19-VB00026	-	-	sensitive
19-VB00027	-	-	intermediate
10-VBH0211	-	-	sensitive
10-VBH0202	-	-	sensitive

* Weak hemolysis after 72 h. ** resistant: growth in 60–80% serum, intermediate: growth in 20–40% serum, sensitive: growth in 0–10% serum.

## Supplementary Materials

The following are available online at https://www.mdpi.com/2076-2607/8/5/739/s1, Table S1: Results of the whole-genome sequence analysis of *Vibrio cincinnatiensis* strains analyzed in this study; Table S2: Minimal inhibition concentrations and resistance profiles of *V. cincinnatiensis* strains; Table S3: SNP distance matrix of *V. cincinnatiensis* strains; Table S4: *V. cincinnatiensis*: Biochemical and phenotypical characterization of veterinary isolates and reference strain NCTC 12012.
