# Supplementary material for: Phenotypic and Genotypic Characterization of Veterinary Vibrio cincinnatiensis Isolates
Source: Microorganisms. 2020 May 15;8(5):739. doi: 10.3390/microorganisms8050739 (PMC7285037; doi:10.3390/microorganisms8050739)
Supplement: Supplementary file 1 [file microorganisms-08-00739-s001.zip › Table S4.docx]

**Table S4:** *V. cincinnatiensis*: Biochemical and phenotypical characterization of veterinary isolates and reference strain NCTC 12012. The German human isolate
19-VB00020 is listed separately.

| **Characteristic** | **V.cincinnatiensis*** | | **19-VB00020** | |
| --- | --- | --- | --- | --- |
|  | **No. of isolates (%)** | |  | |
|  | **+** | **-** |  |  |
| Oxidase | 10 (100) | 0 (0) | pos |  |
| Nitrate reductase | 10 (100) | 0 (0) | pos |  |
| Arginine dihydrolase | 0 (0) | 10 (100) | neg |  |
| Lysine decarboxylase | 0 (0) | 10 (100) | pos |  |
| Ornithine decarboxylase | 0 (0) | 10 (100) | neg |  |
| ONPG (ß-Galactosidase) | 8 (80) | 2 (20) | pos |  |
| Urease | 0 (0) | 10 (100) | neg |  |
| Voges-Proskauer reaction | 0 (0) | 10 (100) | neg |  |
| H_2_S | 0 (0) | 10 (100) | neg |  |
| Indole | 0 (0) | 10 (100) | neg |  |
| **Utilization of:** |  |  |  |  |
| L-Arabinose | 9 (90) | 1 (10) | pos |  |
| D-Glucose | 10 (100) | 0 (0) | pos |  |
| *myo*-Inositol | 8 (80) | 2 (20) | pos |  |
| Maltose | 8 (80) | 2 (20) | pos |  |
| D-Mannitol | 10 (100) | 0 (0) | pos |  |
| D-Mannose | 2 (20) | 8 (80) | neg |  |
| Melibiose | 0 (0) | 10 (10) | neg |  |
| L-Rhamnose | 0 (0) | 10 (100) | neg |  |
| D-Sorbitol | 1 (10) | 9 (90) | neg |  |
| Sucrose | 10 (100) | 0 (0) | pos |  |
| Esculine | 9 (90) | 1 (10) | pos |  |
| Citrate | 2 (20) | 8 (80) | neg |  |
| Gelatine | 0 (0) | 10 (100) | neg |  |
| **Growth in 1 % peptone water** |  |  |  |  |
| + 0 % NaCl | 0 (0) | 20 (100) | neg |  |
| + 2 % NaCl | 10 (100) | 0 (0) | pos |  |
| + 6 % NaCl | 9 (90) | 1 (10) | pos |  |
| + 10 % NaCl | 0 (0) | 20 (100) | neg |  |

*Nine veterinary isolates and reference strain NCTC 12012

+positive phenotype, - negative phenotype
